# cGMP-PKG upregulates PGC1α and improves cardiac function in advanced cardiac hypertrophy independently of RGS2

**DOI:** 10.1186/1471-2210-11-S1-O18

**Published:** 2011-08-01

**Authors:** Eiki Takimoto

**Affiliations:** 1 Division of Cardiololgy, Johns Hopkins Medical Institutions, Baltimore, USA

## Background

Enhanced cGMP-PKG signaling by PDE5 inhibitor (sildenafil) ameliorates cardiac mal-adaptive hypertrophy/remodeling. Although Gq inhibition via PKG-RGS2 activation is an essential mechanism for the early stage anti-hypertrophy under pressure-overload (TAC) in mice, the mechanism for the advanced disease stage remains unknown.

## Results

Impaired mitochondrial respiration was observed in advanced stage dilative 9wk TAC hearts, associated with down-regulation of PGC1α, but not in 1-3 wk TAC hearts. Delayed sildenafil treatment (100mg/kg/day) initiated after hypertrophy establishment (1-3 wk TAC) inhibited progression of remodeling up to 9wks, which was associated with restored PGC1α expression and preserved mitochondrial function, but not with RGS2 activation. Mice lacking RGS2 develop exacerbated early hypertrophy (1wk TAC) which is unresponsive to sildenafil, whereas sildenafil treatment initiated after 1wk ameliorated long term (up to 7wk) progression of cardiac remodeling and function, with restored PGC1α expression and improved mitochondrial respiratory function. Importantly, similar findings were obtained in PKG1α leucine zipper knock-in animals, wherein RGS2-PKG interaction is disrupted. In rat neonatal cardiac myocytes (RNCM), 72hr exposure to phenylephrine (Gq agonist, 20µM) down-regulated PGC1α and impaired mitochondrial respiration, both of which were normalized by sildenafil co-treatment (1µM). Over-expression of PKG up-regulated PGC1α in RNCM, increasing mitochondrial respiration, whereas this was not observed when PGC1α was silenced.

## Conclusion

These results suggest enhancing cGMP-PKG signaling by sildenafil improves cardiac energetics by restoring myocyte PGC1α and thereby ameliorates remodeling progression in advanced stage of pressure-overload hypertrophy, independently of RGS2.

